# Changes in Dimensionality and Fractal Scaling Suggest Soft-Assembled Dynamics in Human EEG

**DOI:** 10.3389/fphys.2017.00633

**Published:** 2017-09-01

**Authors:** Travis J. Wiltshire, Matthew J. Euler, Ty L. McKinney, Jonathan E. Butner

**Affiliations:** ^1^Department of Psychology, University of Utah Salt Lake City, UT, United States; ^2^Department of Language and Communication, Centre for Human Interactivity, University of Southern Denmark Odense, Denmark

**Keywords:** dynamical systems, soft-assembly, repetition priming, fractal scaling, dimensionality, self-organization, coordination

## Abstract

Humans are high-dimensional, complex systems consisting of many components that must coordinate in order to perform even the simplest of activities. Many behavioral studies, especially in the movement sciences, have advanced the notion of *soft-assembly* to describe how systems with many components coordinate to perform specific functions while also exhibiting the potential to re-structure and then perform other functions as task demands change. Consistent with this notion, within cognitive neuroscience it is increasingly accepted that the brain flexibly coordinates the networks needed to cope with changing task demands. However, evaluation of various indices of soft-assembly has so far been absent from neurophysiological research. To begin addressing this gap, we investigated task-related changes in two distinct indices of soft-assembly using the established phenomenon of EEG repetition suppression. In a repetition priming task, we assessed evidence for changes in the correlation dimension and fractal scaling exponents during stimulus-locked event-related potentials, as a function of stimulus onset and familiarity, and relative to spontaneous non-task-related activity. Consistent with predictions derived from soft-assembly, results indicated decreases in dimensionality and increases in fractal scaling exponents from resting to pre-stimulus states and following stimulus onset. However, contrary to predictions, familiarity tended to increase dimensionality estimates. Overall, the findings support the view from soft-assembly that neural dynamics should become increasingly ordered as external task demands increase, and support the broader application of soft-assembly logic in understanding human behavior and electrophysiology.

## Introduction

Humans are high-dimensional, complex systems consisting of many components that must coordinate in order to perform even the simplest of activities. For example, what might seem to be a relatively simple behavior of producing a one syllable utterance, can require as many as 70 muscles to produce (Turvey, 2007) and more than seven cortical structures consisting of billions of neurons (Behroozmand et al., 2015). Further, even if these same muscles and cortical structures are recruited, many behaviors, when performed repeatedly, exhibit subtle, yet measureable differences suggesting a flexible, and context-dependent organization to perform particular tasks (Kello and Van Orden, 2009; Kloos and Van Orden, 2009; Holden et al., 2011). But, the question remains: how do systems with many components coordinate to perform specific functions while also exhibiting the potential to re-structure and then perform other functions as task demands change (Kello and Van Orden, 2009)?

In the movement sciences, attempts to answer this question have typically been described in terms of synergies (Turvey, 2007). The term *synergy* implies that certain components of the system form a temporary grouping capable of performing specific functions (Kelso, 1997, 2009; Tschacher and Haken, 2007). For example, a number of the muscles involved in producing an utterance are also involved in mastication, but there are discrepancies in muscular recruitment between the two tasks. In this way, the particular components of the system are recruited for functionally specific purposes, but they maintain the ability to change as tasks and/or contexts change. This notion is precisely what is meant by suggesting that synergies are formed through *soft-assembly* mechanisms. The term is primarily meant as a contrast to systems that are *hard-molded* where components of the system are rigid and can only perform fixed functions (e.g., Kello and Van Orden, 2009; Anderson et al., 2012). The concept of synergies in this fashion has had a pervasive, although primarily conceptual, history in physiology (Sherington, 1906; Bernstein, 1967; Turvey, 1977) and has even been argued as the key means for linking brain and behavior (Kelso, 2009).

In recent decades, physiologists and neuroscientists have increasingly viewed the brain in similar terms, particularly through the use of concepts from dynamical systems theory (e.g., Stam, 2005; Rodríguez-Bermúdez and García-Laencina, 2015). However, while it would seem that the soft-assembly formulation might be both apt and intuitive to neuroscientists (i.e., neural networks flexibly coordinate in response to task demands; Jensen and Colgin, 2007; Buzsáki, 2010), to our knowledge, this line of reasoning has primarily remained in the explanation of behavior and has not yet been explicitly articulated, nor operationalized in terms of candidate neurophysiological markers.

### Candidate markers of soft-assembly

One candidate marker of soft-assembly is the presence 1/f scaling relations or statistical fractals in time series data (Van Orden et al., 2005). Fractal scaling relations and soft-assembled dynamics can exist without each other (Kello and Van Orden, 2009). But, the presence of scaling relations is nevertheless consistent with an underlying soft-assembly dynamic. In addition, while not a necessary relationship, observations of 1/f scaling relations are often interpreted as reflecting a state of *self-organized criticality*; or a balance of system constraints that is highly-sensitive to context and functionally efficient (Bak, 1996; Van Orden et al., 2003). This is also consistent with the soft-assembly logic. Not only are these fractal patterns found in behavioral data (e.g., Holden et al., 2011), but they are also quite pervasive in physiological measures. For example, fractals in heart beat dynamics have been a useful differentiator between physiological states such as sleep and wakefulness, as well as different states of pathology and aging (Ivanov et al., 1996, 1999a,b; Amaral et al., 1998; Goldberger et al., 2002). In the more general sense, scaling relationships in biological systems are often interpreted as an indicator of healthy and efficient functioning (Goldberger and West, 1987; Bassingthwaighte et al., 1994; Peng et al., 1995a; Van Orden, 2007). Note, however, that while 1/f scaling is one of the most common measures for suggesting that a system exhibits criticality, it can also emerge from other sources (Ivanov et al., 1998; Valverde et al., 2015).

Another candidate marker of soft-assembly is derived from the correlation dimension (D_2_; Rodríguez-Bermúdez and García-Laencina, 2015). The correlation dimension is an indicator of the dimensional complexity of a system with regards to its topological organization. By unfolding an EEG signal into its state space, evaluation of its correlation dimension reveals how complex the organization of the signal is (Pritchard and Duke, 1995). In turn, if a system relies on soft-assembly mechanisms, it should exhibit *dimensional compression*, which is a task-related reduction in the total possible degrees of freedom to the necessary active degrees of freedom (Kay et al., 1987). Indeed, the degrees of freedom upon which a system is functioning is analogous to the dimensionality of the system. Therefore, we can expect that during more neutral states where intrinsic dynamics dominate (e.g., a resting state), a system will have higher dimensionality, and that during task states, the dimensionality should be reduced. This phenomena has been shown in human motor control (Mitra et al., 1998) and has been postulated to also apply to human dialog and interaction (Fusaroli et al., 2014).

### Prior research on potential soft-assembly markers

Following from seminal work in this area (Pritchard and Duke, 1995; Linkenkaer-Hansen et al., 2001), a number of electroencephalography (EEG) studies (Linkenkaer-Hansen et al., 2004; Smit et al., 2013) and work in other neurophysiological modalities (He et al., 2010; He, 2011) have now linked either scaling relations or the correlation dimension (Rodríguez-Bermúdez and García-Laencina, 2015) to various functional states or clinical disorders (Hardstone et al., 2012). However, while this literature has clearly established the relevance of these phenomena to human brain functioning, most of the extant work would be considered as showing necessary but not sufficient evidence for self-organization (Van Orden et al., 2003) and soft-assembly in the brain (Kloos and Van Orden, 2009).

In contrast, in line with the aforementioned theory, more compelling evidence for soft-assembly should take the form of task-related *changes* to fractal scaling relations and dimensionality. However, while it is central that such system metrics should change in relation to task demands (Mitra et al., 1998), it is somewhat rare within neurophysiology to examine this explicitly (Molnar et al., 1995; He, 2011). Even in such cases, it is typical for studies to examine only a small number of electrodes or to adopt analytic procedures that do not account for interdependencies of multiple observations and measurements per participant. In addition, while dynamic approaches to neuroscience are becoming increasingly sophisticated (Palva et al., 2013; He, 2014), in general, studies that apply these methods often face ambiguities about the direction and magnitude of expected effects under various conditions.

### Present study

For all the reasons above, this study aimed to advance and better integrate these literatures by testing specific, directional predictions, informed by the logic of soft-assembly, about the effects of task–related changes to dynamical characterizations of EEG signals. Whereas most prior work has examined either extended time series or task-related activity vs. resting states, by focusing on event-related *change* relative to spontaneous activity, the present study will help address an important gap in the literature. In particular, the findings should inform more detailed predictions regarding experimental effects on the present set of dynamic neural measures and how these measures characterize brain states (Stam, 2005). Additionally, given the large literature implicating event-related alpha dynamics in task-related processing, and the generation of ERPs (Makeig et al., 2002; Hanslmayr et al., 2007; Klimesch et al., 2007; Sauseng et al., 2007), and the potentially complicating effects of narrow-band oscillations on estimated scaling exponents (Hu et al., 2001; Perakakis et al., 2009; Kelty-Stephen et al., 2013), the relationship between alpha oscillations and fractal scaling will also be explored.

To those ends, we used repetition priming to assess evidence for changes in D_2_ and fractal scaling of human EEG during stimulus-locked ERPs both as a function of stimulus onset and familiarity, and relative to rest. Further, we estimated each of these measures for all electrodes and accounted for their interdependence using multilevel modeling. Because soft-assembly dictates dimensional compression, we hypothesized a reduction in the D_2_ between rest and pre-stimulus activity, with a further reduction following stimulus onset, followed by smaller decreases with greater stimulus familiarity. Given that decreases in dimensionality reflect the system becoming more ordered, we in turn hypothesized that scaling exponents should increase between rest and pre-stimulus activity, with further increases following stimulus onset, and with increasing familiarity. Finally, we explored the potential relevance of event-related changes in alpha dynamics with regard to the obtained results, and provide an initial account of the potential physiological relevance of the changes in the various markers.

## Materials and methods

### Participants

Fourteen university students (ages 22.36 ± 3.48, range: 18–31; 9 female) participated in the experiment for course credit and provided informed consent prior to participating. The study was conducted in accordance with ethical standards detailed in the 1964 Declaration of Helsinki and was reviewed and approved by the University's Institutional Review Board. No participants reported any history of diagnosed neurological or psychiatric illnesses and no current psychoactive medication use.

### Repetition priming and resting tasks

Participants were seated in a dimly lit room for EEG acquisition, and completed a 3.5 min eyes-open resting task prior to the repetition priming experiment. For the resting task, participants were instructed to sit comfortably and maintain their gaze on a location in front of them without straining. The priming task then consisted of participants being shown a series of black and white line drawings of common, everyday objects (e.g., animals, tools, articles of clothing, musical instruments, etc.) and being asked to make a decision regarding whether or not the real-life version of the object would fit inside of a shoebox. The images were randomly selected from the Center for Research in Language's International Picture Naming Project (Bates et al., 2003) such that every participant saw a different set of images. Each participant viewed a set of 100 pictures each a total of six times. One hundred novel, non-repeated stimuli were also inserted randomly throughout each participant's stimulus set to facilitate vigilance during the task. Altogether, each participant completed a total of 700 trials (across repetitions) involving exposure to 200 individual stimuli. Each trial began with a fixation cross which was displayed for a randomly variable interval of between 750 and 1,500 ms, followed by the presentation of a line drawing. The image then remained onscreen for 250 ms, after which participants indicated their decision by pressing a button with their right index finger as quickly as possible. The response options, yes and no, were counter-balanced across participants. The entire task lasted ~20 min, and participants were given the opportunity to take a break every 75 trials.

### EEG data acquisition

Sixty-four channels of EEG data were acquired via the ANT-Neuro amplifier system (Advanced Neurotechnology; Enschede, The Netherlands), using Ag/AgCl electrodes mounted in Waveguard caps, arranged according to the 10-5 system (Oostenveld and Praamstra, 2001). Data were referenced online to the average of all active unipolar electrodes (64 in all cases), which was maintained offline. Electrode AFz was the ground. Data were sampled online at 1,024 Hz with no high-pass filter and a digital FIR low-pass filter at 240.8 Hz. This filtering scheme enabled preservation of low gamma range frequencies (i.e., <100 Hz; Jensen et al., 2007), which was important for obtaining accurate estimates of the D_2_ (Pritchard and Duke, 1995). All electrode impedances were maintained below 25 kΩ. Vertical and horizontal eye movements were monitored via two bipolar electrodes placed above and below the left eye (VEOG), and at the external canthi of each eye.

### EEG data processing

Data were loaded into ASA-lab software (ANT Neuro; version 4.9.1) for visual inspection and cleaning. A high-pass filter at 0.1 Hz was applied to facilitate calculation of ERPs. Although, it is generally recommended to perform detrended fluctuation analysis (DFA) on extended time-series for the purposes of characterizing any filter integration effects on the obtained scaling exponents (Hardstone et al., 2012), since the present study is focused on *change* across conditions and all conditions were filtered identically, any such effects should only alter the estimated fractal scaling exponents themselves, rather than comparisons between them. Channels exhibiting significant drift or other noise were interpolated via the spherical spline method implemented in ASA. Representative ocular artifacts were visually identified from VEOG channels and removed using the topographical PCA-based method implemented in ASA-lab (Ille et al., 2002). The continuous data were then epoched from −1,000 to 1,000 ms relative to the onset of the stimulus for the priming task, and in analogous 2,000 ms intervals for the resting data.

Epochs were then manually inspected to exclude artifactual trials and loaded into MATLAB (version R2012b; Natick, MA) for further processing via custom routines. Epochs were then individually linearly detrended and an automated script rejected any remaining trials containing voltages exceeding an absolute threshold of 100 or 75 μV between successive averaged windows of 100 ms. The lowest common denominator of the remaining trials was calculated across the six stimulus exposure conditions, and a random sample of that number of epochs was then taken across conditions (71.86 ± 14.26, range: 44–91). This procedure served to equalize trial counts across conditions within subjects, and also mitigates the potential that systematic stimulus-lag effects (Henson et al., 2004) could influence the calculation of D_2_ or scaling exponents.

**Figure 1:**
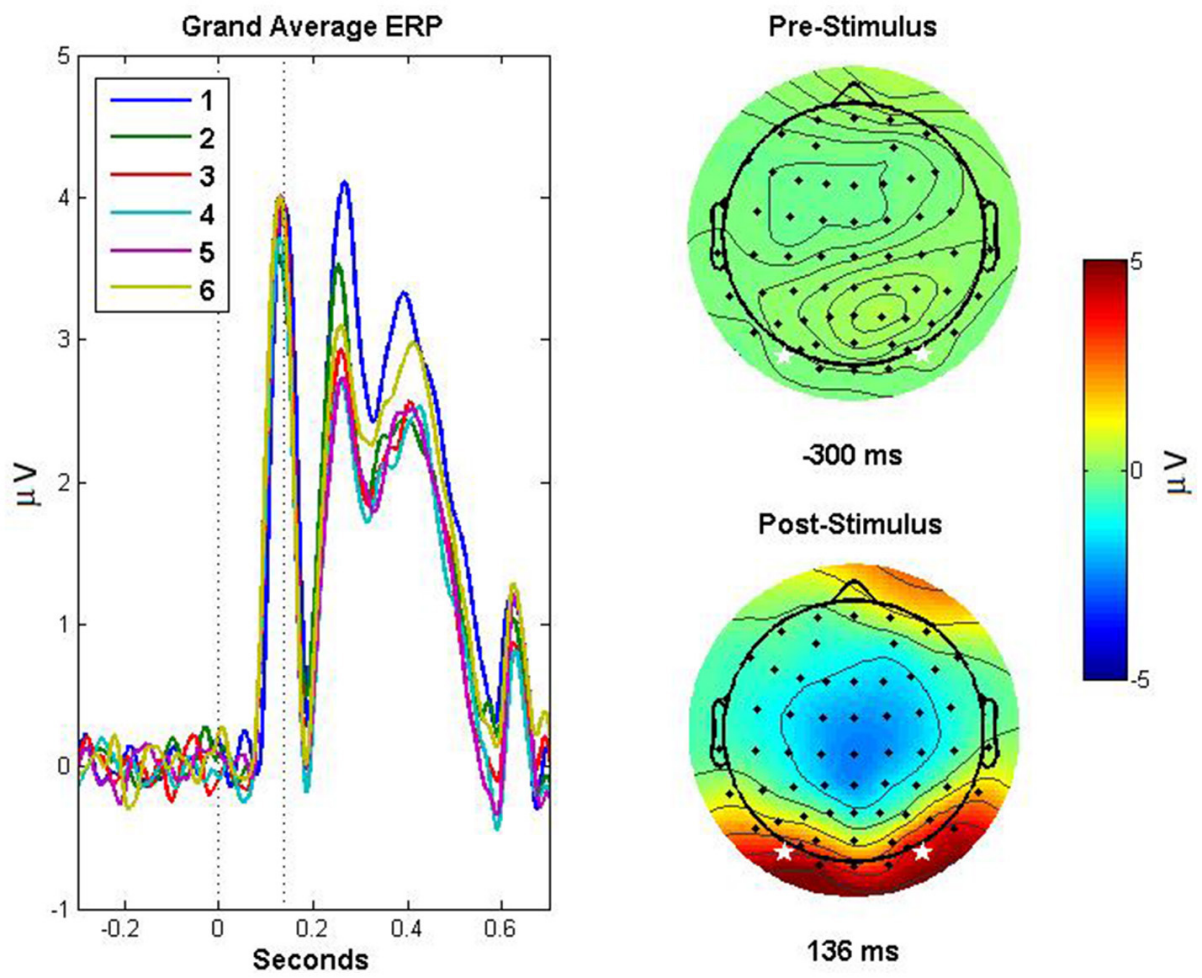
Event related potentials demonstrating repetition suppression effect and scalp topography. ERPs based on the average of channels PO7 and PO8, for each exposure (1–6) to the stimuli, are depicted in the left panel. The dotted lines indicate (from left to right) the onset of the stimulus and the peak amplitude of the grand-average ERP across exposures, corresponding to the P1 component. The right panels depict topographic plots for pre- (top right) and post-stimulus (bottom right) activity, with the stars indicating the electrodes that were averaged to obtain the ERPs depicted in the left panel. The post stimulus scalp map depicts the activity 136 ms post stimulus, corresponding to the peak amplitude in the grand average ERP.

Trials were then averaged within conditions to generate ERPs. No baseline correction or filters were applied to the ERPs for use in calculating D_2_ or scaling exponents. For the purposes of conventional ERP analysis and visualization (see Figure 1), those same epochs were baseline corrected by subtracting the average value 100–300 ms prior to the stimulus onset, and low-pass filtered at 30 Hz for electrodes PO7 and PO8, which were averaged together. The latency of the P100 ERP component (P1) was calculated as the time (relative to stimulus onset) of the maximum value in the first 200 ms, with the mean amplitude of the P1 calculated as the average across 10 ms on either side of the peak. The N1 was calculated in a similar manner, as the minimum value after the P1, but before the period 200 ms post-stimulus. The P2 was likewise calculated, as the maximum value following the N1, but before the period 400 ms post stimulus. Reaction time was calculated as the difference between the onset of the stimulus and the participant's manual response. The EEGlab function topoplot.m was used for visualizing scalp maps (Delorme and Makeig, 2004). For the resting data analyses, two random 2-s epochs were selected from the first and second half of the full 3.5 min resting interval, each of which was further divided into first and second halves (1,000 ms each) for perfect congruence with ERP sampling times. The distinction between the early and late portions of the resting interval and the respective sub-segments allowed for examining differences within the overall resting state data, and also within each of the sampled resting epochs themselves.

In order to evaluate the potential contribution of alpha oscillations to the estimated scaling exponents (i.e., due to concerns about “cross-overs” wherein a dominant oscillation alters the slope the scaling exponent, thereby invalidating the meaningful interpretation of a single scaling exponent; Peng et al., 1995b; Perakakis et al., 2009), or of alpha-desynchronization (ERD; Pfurtscheller and Lopes Da Silva, 1999) to changes in fractal scaling, alpha power and task-related power change (TRPC) were calculated for all conditions. Specifically, the FFT was applied to the Hann-tapered pre- and post-stimulus activity from every single trial. The resulting spectra were squared to obtain power and then averaged over trials to improve the reliability of the estimates. Alpha power was then quantified as the average power from 8 to 13 Hz (Klimesch, 1999) in each condition for the pre- and post-stimulus activity. Resting alpha power was calculated in a similar manner by taking the first half (1,024 samples) of each resting epoch, and then tapering, obtaining power and averaging over trials and then within alpha band. Finally, TRPC, which provides a measure analogous to alpha event-related synchronization/desynchronization (ERS/ERD), was obtained by taking the difference between the log-transformed post- and pre-stimulus alpha values (and analogously for the pre-stimulus and resting alpha values) in a manner similar to that of Benedek et al. (2014).

### Correlation dimension

We calculated the correlation dimension (D_2_) using the corrDim function in the R nonlinearTseries package (Garcia and Sawitzki, 2015) on each stimulus-locked ERP, at each of the 64 electrodes, for each of the six familiarity conditions, prior to and following the stimulus onset. ERPs were 2,048 samples long with the 1,025th sample being the stimulus onset. There were a total of 768 task-related D_2_ estimates per participant. We also estimated D_2_ for each of the four resting state intervals.

The R package we used relies on the Grassberger-Procaccia algorithm (Grassberger and Procaccia, 1983) to calculate D_2_ and we followed the guidelines set by Pritchard and Duke (1995) for calculating D_2_ on EEG data (as detailed below). While explicit details and formulas for this calculation can be found elsewhere (Grassberger and Procaccia, 1983; Jeong et al., 1998), we briefly describe the process here. Estimation of D_2_ involves expanding each time series into a phase space that corresponds to where the data move over time (Takens, 1981). Phase space reconstruction involves the selection of an appropriate tau (delay) and embedding dimension. In order to determine the optimal values for these parameters, we used the estimateEmbeddingDim and timeLag functions in the nonlinearTseries package (Garcia and Sawitzki, 2015). The appropriate tau was determined by taking the first minimum of the averaged mutual information function and the embedding dimension was estimated using the averaged false neighbors method (Cao et al., 1998). We allowed tau to have a maximum value of 60 and the embedding dimension to have a max value of 15. Thus, each D_2_ estimate was calculated using the optimal parameters for that ERP and avoided known issues with parameter selection detailed by Pritchard and Duke (1995). Additionally, we specified a regression range to estimate the linear scaling region across radii with values from 0.01 to the first value of 0.99 to avoid issues with a maximization at values of one that remain constant (Pritchard and Duke, 1995). We also used the Theiler correction to ensure pairs of points that are highly temporally correlated are excluded (Theiler, 1986).

Given proper unfolding of the time-series into phase space, D_2_ is calculated in two primary steps. A correlation integral is calculated by taking the trajectory of points in the phase space and using a radius (r) around each pair of points to calculate the relative number of point pairs that are separated by a distance less than the radius. The next step is to estimate the linear scaling relation as the radius limit goes from zero to infinity for the log of the correlation integral over the log of the radius. The D_2_ estimate indicates how many variables are required to described the system (Chae et al., 2004) and thus, changes in D_2_ indicates whether the system requires more or fewer variables to describe it.

### Detrended fluctuation analysis

We calculated the fractal scaling exponents using the DFA function in the R nonlinearTseries package (Garcia and Sawitzki, 2015) that takes advantage of signal summation conversion to handle both fractal Gaussian noise (fGn) and fractional Brownian motion (fBm; Eke et al., 2000) on each stimulus-locked ERP, at each of the 64 electrodes, for each of the six familiarity conditions, prior to and following each stimulus onset point. We used an ERP signal with length 2,048 in which the 1,025th sample was the stimulus onset. There were a total of 768 task-related scaling exponent estimates per participant. We followed the guidelines for utilizing DFA on EEG data specified by Hardstone et al. (2012) and for time domain fractal analysis, more generally (Eke et al., 2012). This included the pre-processing of the EEG signals detailed above while also taking into consideration appropriate window size, time-series length, and verification of results by comparison with exemplar noise types (i.e., white, pink, and brown), both with and without signal summation conversion (Eke et al., 2000). Additionally, as a validation of applying our analyses to ERP data, which could be influenced by event-related oscillations (Chen et al., 2005; Perakakis et al., 2009; Schmitt et al., 2009) we systematically assessed the potential influence of alpha oscillations on our scaling exponents (see Relation of Alpha Power to Scaling Exponents and Dimensionality Section).

DFA involves removal of the linear trend from a signal for increasing larger window sizes upon which the average fluctuation per window is calculated. The averaged fluctuation per window size is then plotted in log-log metric. A line of best fit for the log-log plot is then calculated. We set a range of window sizes from 4 to 256 samples to estimate the linear scaling relationship from a series of 252 points^1^. The slope is an estimate of the scaling exponent (α) where estimates of 0.5 are known to exhibit white noise that characterizes a random process, 0.5 < α < 1 are known to exhibit fractal long-range temporal correlations in the form of pink noise, and α > 1 corresponds to Brownian noise (fBm) that is temporally correlated although increasingly non-stationary (Eke et al., 2012). We also estimated the α exponents for each of the resting state intervals.

### Analytic strategy

As preliminary steps, we analyzed the effect of repeated stimulus exposure on reaction times (RT) and the averaged ERP amplitudes and latencies of the P1, N1, and P2 components using a repeated-measures ANOVA. We then conducted a series of multi-level models in SPSS Mixed 21 to investigate the effects that the stimulus onset and familiarity had on the estimated scaling exponents and correlation dimensions while accounting for variability due to electrode location. Multilevel modeling is designed to specifically handle nested data sets such as having 64 electrodes per participant (Cohen et al., 2013). For all analyses, the D_2_ estimates and α exponents were treated as dependent variables in the separate models. The stimulus onset was dummy-coded to indicate whether or not the given estimates were prior to, or following the stimulus onset. Familiarity was coded with values increasing from 0 to 5 corresponding to the six levels of familiarity participants encountered in the priming task. Stimulus onset and familiarity were included in the models as fixed effects and we allowed for the interaction of these two variables. We also allowed for a random intercept and random effect for stimulus onset that captured the variability due to electrode (i) for a given individual (j). An example level 1 equation of this model is shown below where D_2ji_ represents the estimate of the correlation dimension at a given electrode (i) for a given individual (j). Generally, β estimates represent either the overall intercept or level 1 estimates of the slope, γ estimates represent the level 2 slope coefficients, and ω estimates represent the error terms associated with deviance from the overall intercept. Additional details of these common parameters and multilevel modeling methods, more generally, can be found in a number of texts (e.g., Cohen et al., 2013; Tabachnick and Fidell et al., 2013; Page-Gould, 2016) The equations are the same for the scaling exponents with replacement of D_2ji_ with α_ji_.

$$\begin{array}{lll} {\text{D}}_{2\text{ji}} & = & \beta_{0\text{i}}+\beta_{1\text{i}}\;{\text{StimOnset}}_{\text{ji}}+\beta_{2\text{i}}\;{\text{Familiarity}}_{\text{ji}}\\ +\;\beta_{3\text{i}}\;{\text{StimOnset}}_{\text{ji}}\cdot \;{\text{Familiarity}}_{\text{ji}}\;+\;{\text{e}}_{\text{ji}} \end{array}$$

Where the level 2 equations are:

$$\begin{array}{lll} \beta_{0\text{i}} & = & \gamma_{00}+\omega_{0\text{i}}\\ \beta_{1\text{i}} & = & \gamma_{10}+\omega_{1\text{i}}\\ \beta_{2\text{i}} & = & \gamma_{20}\\ \beta_{3\text{i}} & = & \gamma_{30} \end{array}$$

Additionally, to examine the influence of alpha power, we ran several diagnostic analyses and two models. The models were similar to those described above, however, now change in scaling exponents (Post-stimulus α – Pre-stimulus α) was used as the outcome variable and TRPC of the alpha band was included as a level 1 predictor, along with the familiarity condition, and an interaction term between the two.

## Results

### Reaction times

Reaction times were analyzed with a repeated measures ANOVA with two within—subject factors, familiarity (Six levels: Exposures 1–6) and response (Two levels: Yes and No), with all reported values using the Greenhouse-Geisser correction for violation of sphericity. Table 1 depicts the means and standard deviations of all RT and ERP measures, as well as D_2_ and α. There was a significant main effect of familiarity [*F*_(1.814, 23.84)_ = 32.075, *p* < 0.001, η_*p*_^2^ = 0.712], such that the median RT decreased with increasing stimulus exposures. There was no significant main effect of response [*F*_(2.932, 38.114)_ = 1.415, *p* = 0.254, η_*p*_^2^ = 0.098], nor a significant interaction [*F*_(1, 13)_ = 0.253, *p* = 0.623, η_*p*_^2^ = 0.019]. *Post-hoc* comparisons for the familiarity effect were Bonferroni corrected, and revealed a pattern where RT in Exp1 were significantly different than all other conditions (highest *p* = 0.001), Exp6 was significantly different from all conditions (highest *p* = 0.001) except for Exp5 (*p* = 0.550), Exp4 and Exp5 were only significantly different from one another (highest *p* = 0.009), and no other differences were significant. Since, as expected, there were no significant differences in RT between yes and no responses, this factor was collapsed for all subsequent analyses.

**Table 1:** Descriptive statistics for all dependent variables.

	**Rest**	**Exp1**		**Exp2**		**Exp3**		**Exp4**		**Exp5**		**Exp6**	
		**Pre**	**Post**	**Pre**	**Post**	**Pre**	**Post**	**Pre**	**Post**	**Pre**	**Post**	**Pre**	**Post**
D_2_	4.88 (0.66)	4.81 (0.7)	4.36 (0.74)	4.78 (0.67)	4.41 (0.79)	4.81 (0.66)	4.45 (0.75)	4.84 (0.65)	4.51 (0.79)	4.82 (0.69)	4.47 (0.74)	4.89 (0.68)	4.47 (0.77)
α	1.01 (0.14)	1.1 (0.18)	1.28 (0.19)	1.11 (0.17)	1.28 (0.18)	1.1 (0.16)	1.28 (0.18)	1.1 (0.17)	1.28 (0.18)	1.1 (0.17)	1.28 (0.18)	1.09 (0.17)	1.28 (0.19)
RT	–	–	904.46 (114.86)	–	809.13 (138.0)	–	792.38 (125.14)	–	769.00 (99.08)	–	749.05 (91.67)	–	735.82 (102.62)
P1-Lat	–	–	129.30 (19.36)	–	123.37 (18.74)	–	127.34 (14.04)	–	122.95 (17.97)	–	121.48 (19.41)	–	124.97 (17.51)
P1-Amp	–	–	5.42 (3.21)	–	4.89 (3.04)	–	5.00 (3.28)	–	4.85 (2.75)	–	5.24 (3.11)	–	5.1 (2.97)
N1-Lat	–	–	173.37 (8.2)	–	172.61 (93.36)	–	172.81 (8.68)	–	172.58 (9.05)	–	173.55 (8.7)	–	172.45 (8.45)
N1-Amp	–	–	0.24 (3.62)	–	0.18 (4.03)	–	−0.14 (4.15)	–	-0.48 (4.12)	–	−0.23 (4.31)	–	−0.14 (4.13)
P2-Lat	–	–	265.53 (52.1)	–	276.06 (58.37)	–	260. (53.48)	–	283.39 (54.68)	–	276.53 (64.54)	–	265.76 (68.02)
P2-Amp	–	–	4.94 (4.0)	–	4.09 (3.81)	–	3.93 (3.62)	–	3.63 (3.09)	–	3.74 (3.26)	–	4.14 (3.42)

*Values are in the format M (SD); Exp, exposures 1–6*.

### ERP amplitudes and latencies

The latency and mean amplitudes of the P1, N1, and P2 were each analyzed with repeated-measures ANOVAs with one factor (familiarity; Exposures 1–6) with all tests corrected for violation of sphericity with the Greenhouse-Geisser method. There was not a significant effect of familiarity on the mean amplitude of the P1 [*F*_(3.067, 36.805)_ = 2.009, *p* = 0.128, η_*p*_^2^ = 0.143], nor the latency of the P1 [*F*_(2.785, 33.417)_ = 2.557, *p* = 0.076, η_*p*_^2^ = 0.176]. There was not a significant effect of familiarity on the mean amplitude of the N1 [*F*_(2.013, 24.160)_ = 1.868, *p* = 0.176, η_*p*_^2^ = 0.135], nor the latency [*F*_(2.171, 26.048)_ = 0.669, *p* = 0.533, η_*p*_^2^ = 0.053]. There was a significant effect of familiarity on the amplitude of the P2 [*F*_(2.776, 33.308)_ = 8.052, *p* < 0.001, η_*p*_^2^ = 0.402] such that it decreased with successive stimulus exposures. *Post-hoc* comparisons for the P2 amplitude effect were Bonferroni corrected and revealed that Exp1 was significantly different than Exp3, Exp4, and Exp5 (highest *p* = 0.038), with no other significant comparisons. There was not a significant effect of condition on P2 latency [*F*_(3.093, 37.122)_ = 0.962, *p* = 0.423, η_*p*_^2^ = 0.074].

### Correlation dimension and fractal scaling relations

Results from examining the effects of stimulus onset and familiarity on D_2_ (D_2_ Task Model) and on the scaling exponents (α Task Model) are shown in Table 2. For D_2_ specifically, there was a significant main effect for stimulus onset. As hypothesized, following the stimulus onset, D_2_ decreased. Further, there was a significant main effect for familiarity such that as familiarity increased the dimensionality also increased. There was no interaction between stimulus onset and familiarity. There were significant random effects on the intercept and stimulus onset suggesting that these results varied significantly as a function of electrode location.

**Table 2:** Model results from prediction of correlation dimension and scaling exponent from stimulus onset and familiarity.

**Estimate**	**D_2_ task model**	**α task model**
**FIXED EFFECTS**		
Intercept	4.786 (0.025)^***^	1.103 (0.007)^***^
StimOnset	−0.396 (0.030)^***^	0.259 (0.004)^***^
Familiarity	0.015 (0.006)^**^	−0.001 (0.001)
StimOnset·Familiarity	0.007 (0.008)	0.003 (0.001)^**^
**RANDOM EFFECTS**		
Intercept	0.021 (0.005)^***^	0.035 (0.001)^***^
StimOnset	0.021 (0.006)^***^	0.012 (0.001)^***^

All standard errors are shown in parentheses following the estimate. ^*^p < 0.05;

** p < 0.01;

*** *p < 0.001*.

**Figure 2:**
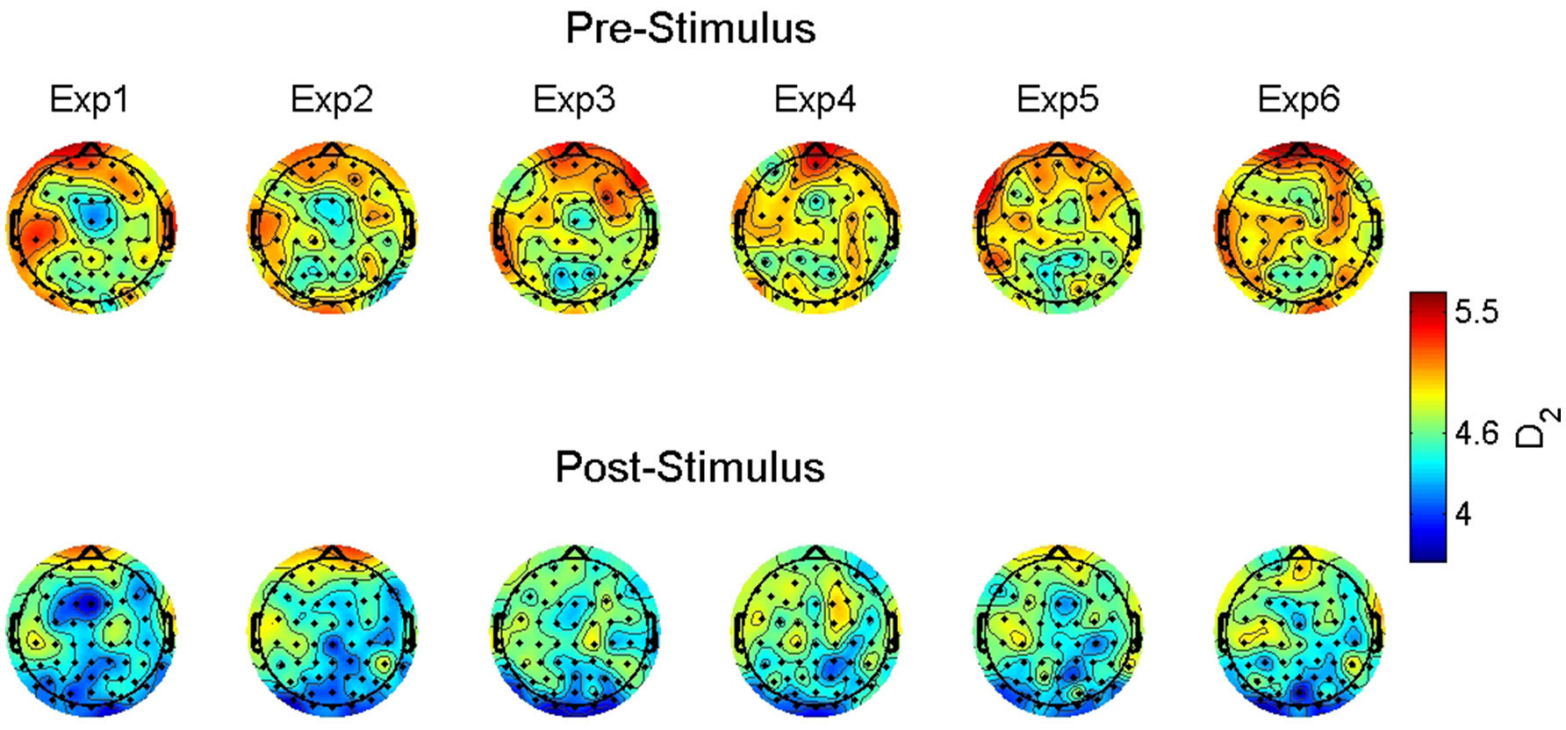
Group-averaged correlation dimension scalp maps. The maps show the estimated correlation dimension (D_2_) across increasing levels of familiarity (left to right) prior to (top) and following (bottom) stimulus onset. Exp = exposure.

Figure 2 shows the observed D_2_ estimates as a function of stimulus onset, familiarity condition, and electrode location to illustrate this observed variability. Given the significant random effects combined with the scalp maps, the observed dimensionality prior to the stimulus onset ranges from 4.6 to 6. However, following stimulus onset the estimates were reduced to a range of approximately below 4 to a max of 4.8.

**Figure 3:**
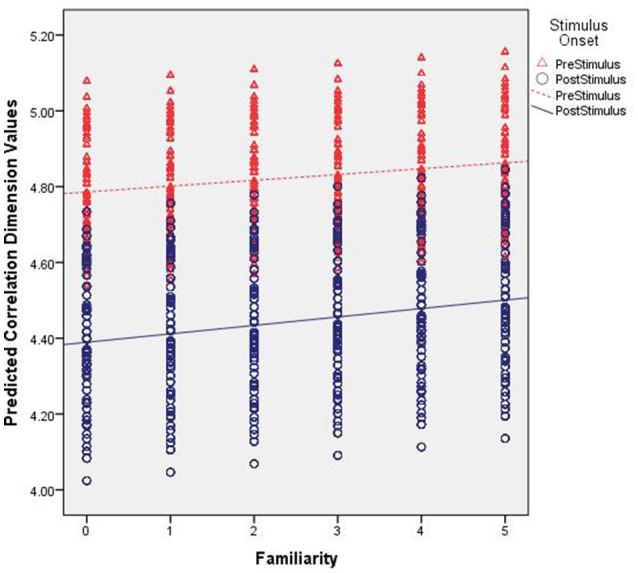
Predicted correlation dimension values. The figure shows the predicted Correlation Dimension (D_2_) values across increasing levels of familiarity (left to right along the x axis) and segmented by pre- (red triangles) and post-stimulus (blue circles).

The D_2_ values predicted by our model are shown in Figure 3. That is, these are the expected values of D_2_ for each electrode given the parameters and variables included in our multi-level model with the best fitting line added for pre- and post-stimulus. Specifically, they illustrate the observed results as a function of familiarity and stimulus onset. First, note the differences in intercepts between pre- and post-stimulus. With no stimulus familiarity, D_2_ is much lower following stimulus onset. As familarity increases, so too do the predicted D_2_ values. However, the slope coefficient for post-stimulus is stronger. While the interaction was not significant, the pattern of predicted values from the model shows that as familiarity increases, the dimensionality following the stimulus onset is converging toward the values of dimensionality prior to stimulus onset, which is also similar to estimates of dimensionality during resting states (described below).

For the scaling exponents derived using DFA, we observed a significant main effect for stimulus onset. There was a general increase in α following stimulus onset. Given the intercept estimate of 1.1 and the 0.26 estimate for stimulus onset, the time series were becoming more fractal, but moving toward brown noise that has some non-stationary drift as well as random-walk properties (see Hardstone et al., 2012). Further, there was no significant effect for familiarity. There was, however, a significant interaction between stimulus onset and familiarity such that following the stimulus onset, there was an overall increase in the exponents that slightly increased further as familiarity increased. Lastly, there were significant random effects for the intercept and stimulus onset suggesting that these results varied significantly as a function of electrode location.

**Figure 4:**
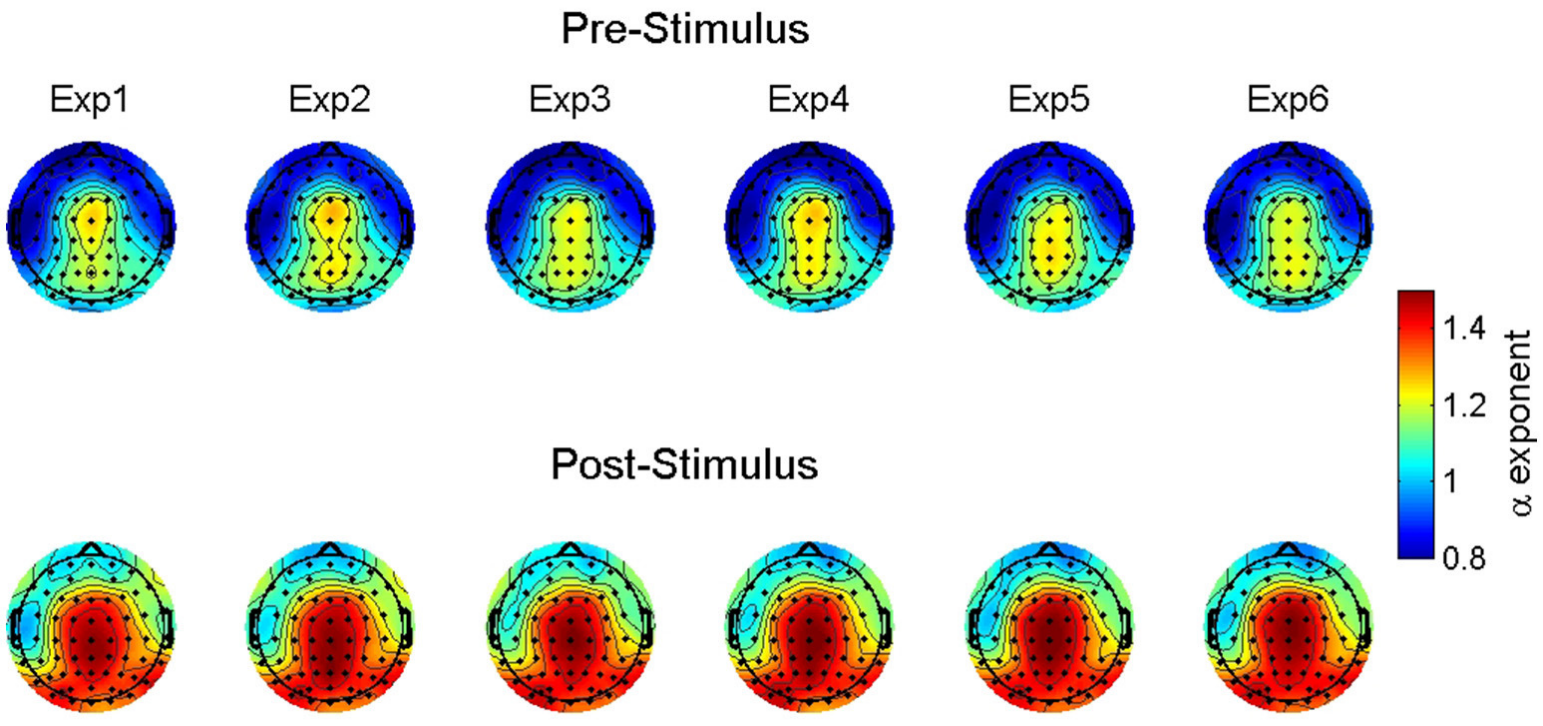
Group-averaged scaling exponent scalp maps. The maps show the estimated scaling exponents (α) across increasing levels of familiarity (left to right) prior to (top) and following (bottom) stimulus onset. Exp = exposure.

Figure 4 shows the observed α exponent estimates as a function of stimulus onset, familiarity condition, and electrode location to illustrate this observed variability. While the overall model suggests an α exponent that was just slightly above 1, the combination of significant random effects and scalp maps of the observed estimates highlights the case that at some locations prior to stimulus onset the α exponents were <1 and thus, falling into the pink noise range. However, following stimulus onset almost all estimates change to a value of 1 or greater.

**Figure 5:**
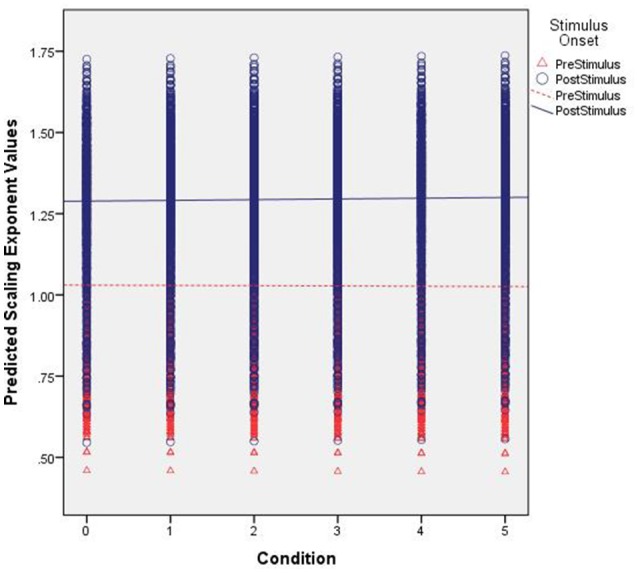
Predicted scaling exponent values. The figure shows the predicted scaling exponents (α) across increasing levels of familiarity (left to right along the x axis) and segmented by pre- (red triangles) and post-stimulus (blue circles).

The α values predicted by our model are shown in Figure 5. First, note the differences in intercepts between pre- and post-stimulus. With no stimulus familiarity, α is lower prior to stimulus onset. As familarity increases, the predicted values of α generally remain constant. However, for the significant interaction effect, whereas the fractal scaling prior to stimulus onset remains largely the same as familiarity increases, the small positive slope coefficient suggests that the post-stimulus scaling is very slightly increasing as familiarity increases. Yet, Figure 5 shows this difference might be quite negligible.

### Resting state comparisons

**Figure 6:**
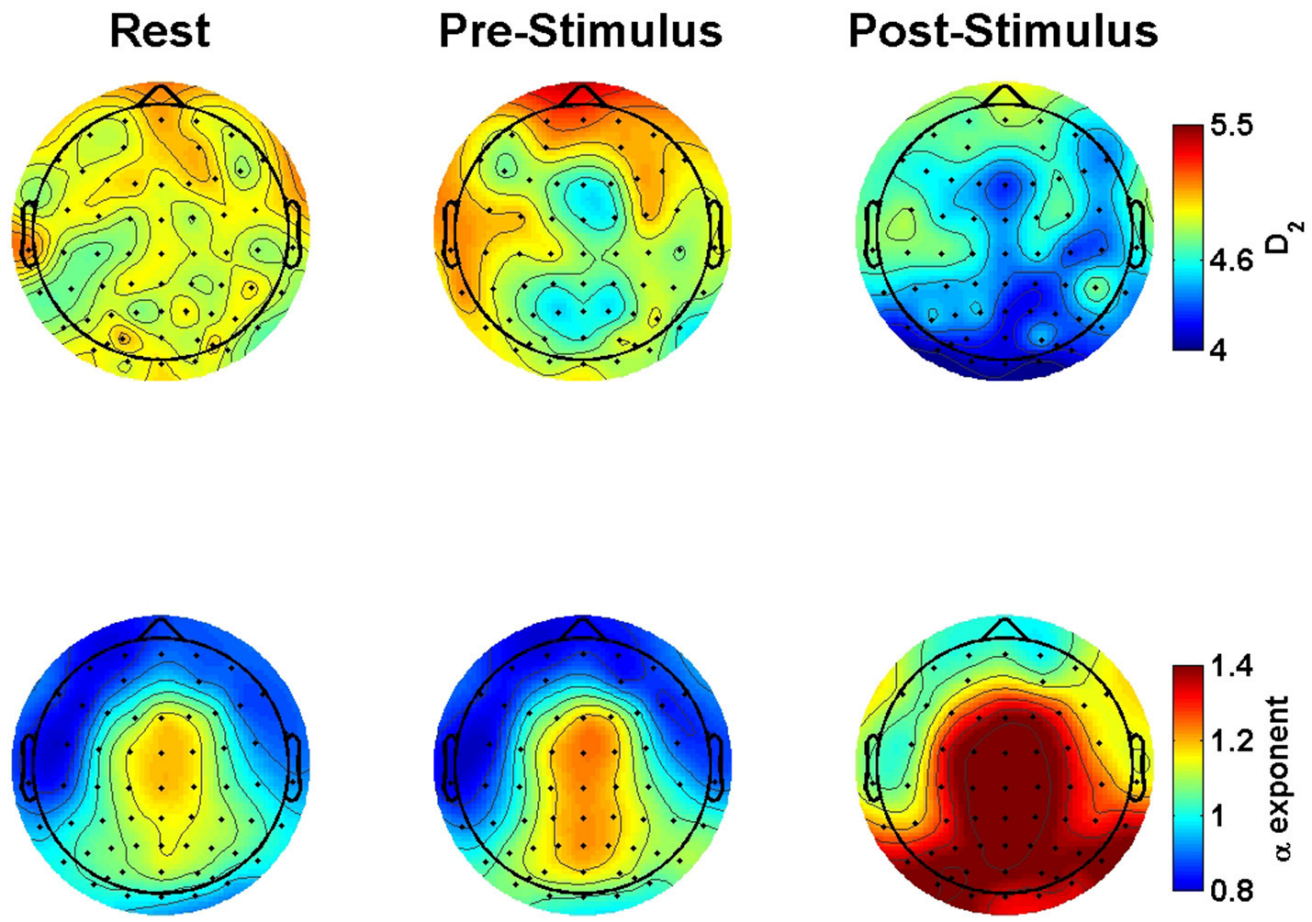
Group-averaged correlation dimension and scaling exponents scalp maps comparing rest to pre- to post-stimulus. The maps show the estimated correlation dimension (top) and scaling exponents (bottom) as a function of resting conditions (left), prior to (middle) and following (right) stimulus onset.

Analyses of the various resting data segments indicated that neither D_2_ nor scaling exponents differed as a function of whether the resting epochs were taken from early or later segments of the recording period, nor whether the first vs. second half of the epoch was analyzed. Thus, we included all rest estimates for comparison with all event-related (i.e., ERP) time-series data, pre- and post-stimulus onset. Table 3 shows the results from a series of models testing these Rest-Task comparisons. Overall, we observed significant effects for rest suggesting that the spontaneous EEG differed not only from the post-stimulus task-related ERP, but also from the pre-stimulus period as well (see Figure 6). D_2_ was significantly higher during rest than during the pre- and post-stimulus task-related ERP. Further, the α exponents were significantly lower during rest than during the pre-stimulus period and the post-stimulus ERP. There were also significant random effects on the intercept for all analyses, suggesting that D_2_ and the α exponent varied significantly as a function of electrode location.

**Table 3:** Model results from resting state EEG compared to task-related ERP.

**Estimate**	**D_2_ Rest-pre-stim. model**	**α Rest-pre-stim. model**	**D_2_ Rest-post-stim. model**	**α Rest-post-stim. model**
**FIXED EFFECTS**				
Intercept	4.824 (0.015)^***^	1.027 (0.015)^***^	4.445 (0.017)^***^	1.294 (0.016)^***^
Rest	0.051 (0.014)^***^	−0.021 (0.003)^***^	0.430 (0.015)^***^	−0.287 (0.004)^***^
**RANDOM EFFECTS**				
Intercept	0.010 (0.002)^***^	0.015 (0.003)^***^	0.013 (0.003)^***^	0.016 (0.003)^***^

All standard errors are shown in parentheses following the estimate. ^*^p < 0.05; ^**^p < 0.01;

*** *p < 0.001*.

### Relation of alpha power to scaling exponents and dimensionality

Given that alpha oscillations are often the dominant oscillatory processes in broadband EEG, their potential effects on scaling exponents and D_2_ warrant particular focus for two reasons. First, some scaling exponents may be affected by cross-overs (i.e., a change in the scaling relationship) due to the sinusoidal nature of alpha band oscillations (Hu et al., 2001; Perakakis et al., 2009; Kelty-Stephen et al., 2013). Second, since alpha oscillations are also subject to the well-established ERD phenomenon involving decreases in alpha power following stimulus onset (Klimesch et al., 2007), it is possible that the observed scaling exponent estimates simply reflect ERD-related changes in the power spectrum as opposed to a genuine shift in the scaling relations. *Post-hoc* analyses were run to address these possibilities^2^.

We observed that average alpha power across all electrodes and conditions was 0.555 μV^2^ (range = 0.028–13.15). For rest, the average was 0.542 *w*/μV^2^ (range = 0.047–6.40). For pre-stimulus, the average was 0.994 μV^2^ (range = 0.042–13.15). Finally, for post-stimulus, the average was 0.663 μV^2^ (range = 0.028–4.58). As a way to examine those instances where alpha power may be atypically high across all electrodes, participants, and conditions, we used modified z-scores >3.5 to determine those alpha power scores that greatly deviated from the median (Iglewicz and Hoaglin, 1993). Overall, we observed that only 1.25% of cases (*N* = 146) exceeded this threshold and, on average, these high alpha power cases were 5.74 μV^2^ (range = 3.89–13.15). Of this 1.25% of cases, ~15.07% were from the resting interval, 82.19% were pre-stimulus cases, and 2.74% were post-stimulus cases. Of this 1.25% of cases, 86% were from parietal, occipital, or parieto-occipital electrodes, 11% were from medial electrodes, and 3% from a fronto-central electrode.

While the prevalence of cross-overs appeared minimal, in order to test their influence on our results, we excluded each of the scaling exponent estimates that exceeded our threshold for having high power. We then re-ran the original model excluding these cases with potentially problematic scaling estimates (Table 4). The overall pattern of results was the same. The only notable, yet negligible, difference is the reduction of the intercept estimate closer to a value of one. Thus, because these results are largely the same, we can conclude that our interpretations based on a single meaningful scaling exponent are generally valid. However, in the very small percentage of localized cases where alpha power was higher, there is likely some multifractal behavior (perhaps, only bi-fractal; cf Kelty-Stephen et al., 2013) being exhibited with two or more distinct scaling relationships contingent upon the scale (i.e., below and above the alpha range).

**Table 4:** Model results comparing scaling exponents with all cases included and with those cases that denoted high alpha power removed.

**Estimate**	**α Task model (Original model)**	**α Task model (High power alpha removed)**
**FIXED EFFECTS**		
Intercept	1.103 (0.007)^***^	1.029 (0.007)^***^
StimOnset	0.259 (0.004)^***^	0.258 (0.004)^***^
Familiarity	−0.001 (0.001)	−0.001 (0.001)
StimOnset·Familiarity	0.003 (0.001)^**^	0.003 (0.001)^**^

All standard errors are shown in parentheses following the estimate. ^*^p < 0.05;

** p < 0.01;

*** *p < 0.001*.

To examine the degree to which alpha ERD may have contributed to the observed changes in scaling exponents, alpha TRPC was included as a predictor in a model predicting change in scaling exponents (See Table 5). Because this variable was computed as post-stimulus minus pre-stimulus alpha power (log transformed), negative values correspond to ERD. Conversely, since fractal scaling exponents increased following stimulus onset, the post-stimulus minus pre-stimulus change variable generally reflects positive values. Thus, if the obtained changes in α scaling exponents are the result of alpha ERD, we would expect a *negative* relationship between the two change variables (i.e., TRPC should inversely predict scaling increases). In contrast to this expectation, results demonstrated a *positive* relationship between alpha TRPC and change in scaling exponents in both rest-to-pre and pre-to post models. Thus, while ERD may occur at some electrode sites (most likely the posterior-occipital sites with higher alpha power), at the whole brain level in the sample as a whole it appears that increases in fractal scaling are associated with increased alpha power.

**Table 5:** Model results from alpha power on task related changes in fractal scaling.

**Estimate**	***Δα* Rest-to-Pre**	***Δα* Pre-to-Post**
**FIXED EFFECTS**		
Intercept	0.101 (0.004)^***^	0.179 (0.004)^***^
Familiarity	−0.004 (0.001)^***^	0.0003 (0.001)
TRPC	0.027 (0.005)^***^	0.023 (0.006)^***^
Familiarity ^*^ TRPC	−0.003 (0.001)^***^	−0.009 (0.001)^***^
**RANDOM EFFECTS**		
Intercept	0.015 (0.001)^***^	0.011 (0.001)^***^

All standard errors are shown in parentheses following the estimate.

* p < 0.05;

^**^p < 0.01;

*** *p < 0.001*.

## Discussion

The soft-assembly metaphor provides a conceptual framework for understanding how systems with many components may flexibly structure themselves in response to shifting task demands (Kello and Van Orden, 2009). In this study, we aimed to test the applicability of soft-assembly logic for characterizing task-related changes in human brain states. Specifically, we investigated changes in fractal scaling exponents and dimensionality estimates obtained from the spontaneous scalp-recorded EEG during a resting state vs. pre- and post-stimulus periods from ERPs recorded during a repetition priming task. We expected to observe systematically increasing fractal scaling exponents and decreasing dimensionality estimates upon stimulus onset and as perceptual familiarity increased. Our results were largely, but not unequivocally, consistent with our predictions. However, we argue that such a pattern of results provides initial support for applying soft-assembly logic in characterizing experimental effects on gross-level neural dynamics.

First, and most importantly, there was an overall increase in scaling exponents and decrease in the correlation dimension from rest to pre- to post-stimulus. These findings indicate that, consistent with our hypotheses, as task demands increasingly constrain a participant's behavioral state, the overall EEG dynamics become increasingly ordered, as interpreted from the soft-assembly perspective. Second, however, effects of stimulus familiarity on our measures did not track expectations in the predicted direction. With regard to our hypothesis that increasing stimulus familiarity would result in greater scaling exponents, there was no effect of familiarity on the scaling exponents. By contrast, although D_2_ did decrease both from rest to the pre-stimulus period and again following stimulus onset, counter to our expectation, familiarity resulted in an increase in D_2_ for both pre- and post-stimulus periods. Although, unexpected, these familiarity effects are unlikely to reflect a failed manipulation because the main effect of familiarity on RT replicated the classic behavioral finding in repetition priming studies (Bentin and McCarthy, 1994), while the main effect of familiarity on P2 amplitude replicated a common repetition suppression effect (Ben-David et al., 2011; Hsu et al., 2014). Thus, the results as a whole link classic repetition priming effects in RT and ERPs to event-related changes in dynamic measures, but suggest the need for a nuanced interpretation of their inter-relations. Taken together, we view these findings as supporting the potential utility of the soft-assembly logic for characterizing experimental neurophysiological effects. We expand on the theoretical basis for this interpretation relative to each of our measures in the following sections.

### Fractal scaling

With regard to the observed changes in fractal scaling, both the physiological and behavioral literatures provide evidence for adaptive, intrinsic coordination in human physiological systems. For example, research on heartbeat dynamics has demonstrated that healthy individuals exhibit long-range temporal correlations in the pink noise range, with a breakdown of these dynamics characterizing various pathological states (Peng et al., 1995a; Goldberger et al., 2002). As noted above, scaling exponents consistent with pink noise have been interpreted as a plausible indicator for a state of criticality within the system (Cannon et al., 1997), with the “resting” human EEG typically also exhibiting scaling relations in this pink noise range (Pereda et al., 1998; Linkenkaer-Hansen et al., 2001; Palva et al., 2013; Euler et al., 2016), including in the present data. Notably, the natural variability exhibited in human behavior has also been shown to exhibit fractal patterns in this range (Van Orden et al., 2003, 2005; Kello et al., 2008), where again the observed long range temporal correlations imply a coordination reflecting the intrinsic system dynamics (Van Orden et al., 2003; Holden et al., 2011). Thus, examination of resting states in physiological studies and variability in behavioral studies primarily capture the system's endogenous variation, and consistently suggest what may be optimized patterns of intrinsic dynamics (cf. Likens et al., 2015).

However, when prompted to complete a behavioral task such as respond to stimuli, the intrinsic behavioral and physiological dynamics of the system are perturbed (Van Orden et al., 2003; Holden et al., 2011). The system then shifts from exhibiting its coordinated, intrinsic dynamics, and fluidly reorganizes to perform the task. On that basis, the observed increases in scaling exponents between periods of rest, pre-, and post-stimulus in the present study are conceptually consistent with this general pattern where task demands perturb the intrinsic electrophysiological dynamics. However, whereas we had predicted a straightforward relationship with increased fractal scaling indicating increased “orderliness” within the system, the results imply the need for a more nuanced interpretation. Specifically, during rest, the scaling exponents suggest a highly persistent and thus, coordinated system exhibiting pink noise (~ α = 1.01), whereas during pre-stimulus periods, the dominant pattern appears as strongly anti-persistent fractional Brownian motion (fBm; ~ α = 1.10). When the coordination shifts from persistent to anti-persistent in this way, it suggests a change from long range positive correlation to long range negative correlation (Delignieres et al., 2011), which although the sign changes, still reflects coherent and coordinated organization. Speculatively, it is interesting to consider whether a transition to persistent vs. anti-persistent coordination reflects the fact that the dynamics are still being “intrinsically” structured in an anticipatory physiological state, but in a way that reflects the direction of neural resources toward external rather than internal events. Following stimulus onset, however, we observed further increases in the scaling estimates (~α = 1.28). Thus, whereas the results indicated that the dynamics became increasingly *fractal* as external demands increased, the further increase toward fBm is not necessarily consistent with increasing *order* within the system.

Consistent with other research, fBm has been observed in a number of reports on task-related brain activity (Buiatti et al., 2007; cf. Vega and Fernandez, 2012; Sleimen-Malkoun et al., 2015). However, in contrast to the present findings, work done by He and colleagues with electrocorticography (He et al., 2010) and functional MRI (fMRI; He, 2011) has typically shown task-related *decreases* in fractal scaling of broadband signals, apparently including from fBM to the pink range, or from the pink to the white noise range. Similarly, for narrow-band electrophysiological signals, Palva et al. (2013) observed non-significantly decreased scaling exponents between rest and threshold-detection tasks (see their Figure S2), while Smit et al. (2013), observed generally increased exponents for finger-tapping vs. rest, albeit with decreased exponents at scalp locations that had previously showed the greatest task-related fractal scaling (see their Figure 3A).

While there are likely many reasons for the inconsistencies within this literature (Hardstone et al., 2012; Smit et al., 2013), variability in the estimated fractal scaling exponents across studies seems to be the norm, and draws attention to outstanding questions for research on scaling relationships (Kello et al., 2010; Holden et al., 2011). Overall, such discrepancies highlight the need to establish predicted patterns of effects according to the various tasks (Vega and Fernandez, 2012), recording modalities (He, 2014), signal processing methods (Kello et al., 2010), measurement contexts (Holden et al., 2011), and analytic strategies employed (Euler et al., 2016). While the present study took a step in that direction (by supporting directional predictions derived from soft assembly), it is notable that not only do those results contrast with other literature, but the empirical exponents themselves (i.e., moving from pink noise to fBm) raise additional questions about their interpretation and significance. Most importantly for present purposes, the most common approach of characterizing scaling exponents obtained from extended resting or task intervals (and thus obtaining metrics that encompass numerous individual events and responses) may not capture the same dynamics as the present event-related approach, thereby possibly explaining our discrepant results. Considering that soft-assembled systems are inherently defined by the fluid and functional recruitment of resources in relation to an event, it follows that event-related approaches may better capture more fine grained aspects of the neurophysiological dynamics.

### Correlation dimension

While D_2_ has previously been used to evaluate the complexity of EEG signals, it has not been explicitly linked to the soft-assembly characterization of neurophysiological dynamics. Our findings are consistent with a seemingly robust phenomenon that states of goal oriented behavior have lower dimensionality relative to resting conditions (Sammer, 1996; Aftanas et al., 1998; Anokhin et al., 1999; but see Lamberts et al., 2000). To our knowledge, only two neurophysiological studies have explored event related changes in D_2_ and found, consistent with the present results, a reduction in dimensionality following stimulus onset (Molnar et al., 1995; Molnar, 1999). However, in that work, a very limited number of electrodes were examined (compared to our dimensionality estimates for every electrode), and dependencies between electrodes were not accounted for. Regardless, even despite the different tasks employed in those studies (infrequent oddball) and the present study (repetition priming), the consistent event-related reduction in dimensionality likely reflects a general feature of stimulus-related processing, rather than a specific effect of certain task requirements.

Task-related reductions in D_2_ could indicate the brain is suppressing task-irrelevant processes to achieve a less complex system, consistent with the finding that meditation also reduced D_2_ (Aftanas and Golocheikine, 2002). Despite recent debates (Shah et al., 2004; Mazaheri and Jensen, 2006; Telenczuk et al., 2010), ERPs have been classically viewed as the linear combination of spontaneous and evoked activity (Arieli et al., 1996). This characterization is consistent with a *hard-molded system* where increasing demands drive the addition of specific processes to the system (Kloos and Van Orden, 2009). As applied to ERPs, we reasoned that a hard-molded system would exhibit task-related increases in D_2_ during the transitions from rest to pre- and post-stimulus activity, as a result of the additive contribution of evoked to spontaneous activity. In contrast, based on our presumption of neural soft-assembly, and fMRI evidence for non-linear interactions of evoked and spontaneous dynamics (He, 2013), we expected to observe task-related reductions.

Although, the results regarding more global task effects reflected such a pattern, our finding that D_2_ increases with familiarity was contrary to our prediction. Specifically, we expected that increasing levels of familiarity with the stimuli, and thus increased skill at the task, would correspond to decreasing dimensionality estimates. As a result, we are left with a puzzling question as to why degrees of freedom would actually increase as the same task is repeated? One plausible interpretation is that the intrinsic dynamics become less and less perturbed in the course of processing the stimuli as participants become more familiar with them. That is, the system may more readily return to the pre-stimulus state following a perturbation by a familiar stimulus, making the post-stimulus time-series as a whole appear less ordered or more “noise-like.” In general, this interpretation would be supported by the fact that the dimensionality of the post-stimulus period trends toward the dimensionality of the rest period as familiarity with the task increases.

That said, as an alternative explanation, prior work has shown that correlation dimension estimates are affected by the presence of noise. While EEG data can never be noise-free, EEG based dimensionality estimates are acceptable as long as they are interpreted in relative terms (Pritchard and Duke, 1995). But, because ERPs specifically aggregate across many observations to improve the signal-to-noise ratio, it is possible that the decrease in ERP amplitude as familiarity increases (i.e., repetition suppression) allows for increased levels of noise to be evident in the signal from trials where familiarity is greater. This could contribute to increased variability during phase space reconstruction and in turn, increased dimensionality estimates. One complication for this interpretation, however, is that we observed only a main effect of familiarity, suggesting that even in the pre-stimulus periods, where there is no ERP amplitude, dimensionality tended to increase as well. This main effect would seem to challenge the above interpretation as well, potentially suggesting a time on task effect on both the pre- and post-stimulus dynamics. Ultimately, future experimental work should examine whether dimensionality increases over increasing levels of familiarity arise from true changes in the task's effect on intrinsic system dynamics, vs. more mundane signal-to-noise effects of repetition suppression, or other possibilities.

### Potential relevance of alpha desynchronization

The influence of pre-stimulus alpha activity on post-stimulus neural activity is a well-documented phenomenon (Haig and Gordon, 1998a,b; Barry et al., 2000), primarily in the form of event-related alpha desynchronization (Hanslmayr et al., 2007; Min et al., 2007). We found a positive relationship between changes in alpha power and changes in scaling exponents. This suggests that increases in scaling exponents and dimensional compression in the brain is related to alpha ERS as opposed to ERD, contrary to expectations. That said, while the present study examined a whole brain approach to soft assembly and alpha dynamics, there is likely task and regional specificity in these effects, which if uncovered, could further integrate the dynamical systems and neurophysiological literatures. Future research could begin to explore these nuances to elaborate whether (and how) neural soft-assembly mechanisms may change under various circumstances.

### Connecting behavioral and neurophysiological perspectives on soft-assembly

A final outstanding question concerns the relation of our present argument for neural soft assembly, to soft-assembly within the behavioral literature, where it has typically been discussed. From a behavioral perspective, our participants completed a task that requires them to exhibit the same behavior repeatedly (i.e., respond with a key press to the stimuli). While the observed reaction times decreased with increasing familiarity, at a molar level, the actual behavior (pressing one key or another) changes very little from trial to trial. In this way, our task is similar to behavioral studies that have examined repetitions of the same behaviors (e.g., uttering the same syllable repeatedly; Kello et al., 2008). Recall that Kloos and Van Orden (2009) posited that a soft-assembled system is characterized by a state of criticality, and that the subsequent recruitment of resources is toward a functional outcome in a fluid and temporary fashion. Across behavioral and neural studies, the presence of fractal scaling relations has been interpreted as an indicator that the intrinsic dynamics are in a critical state and thus, provide an indicator that the system is likely soft-assembled (Kello and Van Orden, 2009). We sought to extend this empirical basis by focusing on changes, not only in fractal scaling due to task demands, but also changes in the dimensionality. However, while we focused on a neurophysiological context, we can see two different ways in which soft-assembly may connect behavior and neurophysiology.

First, soft-assembly implies functional changes to the way a system is organized and coordinated, concomitant with changes in tasks demands. Specifically, the focus of this study has been on this first idea where we argued and evaluated the idea that dimensional compression and increasing order should occur within the neurophysiological system as task demands increase. In our study, the major changes to behavior took the form of participants either resting or performing the task, and we observed corresponding changes in the organization and coordination of the neurophysiological system. While we can connect soft-assembly in neurophysiology to behavior in this way, these kinds of changes can also occur in the behaviors themselves as a function of varying task constraints and task engagement (Likens et al., 2015). So this first notion of soft-assembly is really that the system, in behavior and/or neurophysiology, changes quite fluidly and we should observe these changes in measures reflecting system organization and coordination.

Second, in the most general sense, a soft-assembled system can engage in the same global behavior, but its way of accomplishing it can vary substantially. More technically, this notion implies that a system can re-configure itself to reach a consistent global outcome from anywhere in its state-space, yet its more minute dynamics might importantly vary across different instances of producing the same molar response. In a behavioral context, this type of soft-assembly is observed when a hammer or other tool is wielded repeatedly yielding the same results, but the joints never precisely following the same trajectories (Bernstein, 1967; Biryukova and Bril, 2015). Or, it is also observed when producing the same utterance repeatedly, yet there are differences in the acoustic properties (Kello et al., 2008), as well as when the task is to simply respond to a certain stimulus thousands of times and yet there is meaningful variability in those reaction times (Van Orden et al., 2003). The idea of the system being able to re-configure is supported even further by evidence of rapid compensatory mechanisms, such as studies of speech production where even when unexpected perturbations to the jaw are made, there is no perceivable distortion to the observed speech (Kelso et al., 1984; see also Bardy et al., 2007; Riley et al., 2012; Harrison and Stergiou, 2015). In this way, the observed variability would often be taken as noise (Van Orden et al., 2003) while in fact those different underlying configurations may nevertheless be meaningfully facilitating the same global outcome.

Within neuroscience, we also see evidence in the literature for this view of soft-assembly as different paths to the same global outcome. One example supporting this is Walter Freeman's classic work on electrophysiological responses to olfactory stimuli (Freeman, 1991). There, he demonstrated that while there are detectable consistencies in the observed EEG signals when the same odor is perceived repeatedly, the signals often vary in meaningful ways (e.g., the average amplitude can be higher or lower). Importantly, the observed consistencies, derived from perception of the same stimuli, only account for approximately one to three quarters of the total population activity comprising the signal (Freeman, 1991). Thus, the variability in responding to the same stimulus across repeated presentations can vary as much as, if not more than, it is consistent.

While the latter aspect of soft-assembly was not the focus of the current paper, our results may provide initial and tentative support for it as well. That is, the pattern of results shown in the EEG scalp maps for the correlation dimension may at first be interpreted as noise and inconsistency across levels of familiarity, yet they may nevertheless reflect the soft-assembly of brain activity to reach a consistent outcome. Again, even as participants become more familiar with the stimuli, the task remains the same. Although, we did not test a spatial model (cf. Euler et al., 2016), we can visually observe variability in the dimensional complexity across electrodes. We expect that examining variability in the recruitment of neurophysiological resources, while task demands remain more or less constant (cf. Likens et al., 2015), would be worth examining in future research to show how global outcomes can be reached with varying neurophysiological configurations.

In a more general context, we expect that considering these two aspects of soft-assembly in both behavioral and neurophysiological contexts, as well as relations between the modalities, can aid in substantively advancing this science and addressing the question of how many-component systems can become coordinated across a variety of scales. We have taken a first step toward this end by recognizing that fractal scaling is a necessary, but not sufficient, characteristic of a soft-assembled system, and demonstrating how task demands can influence such scaling relations in a directional and theoretically-consistent way, while also showing complementary changes in dimensionality estimates. Nevertheless, like behavioral research on soft-assembly (e.g., Van Orden et al., 2003), the use of only two markers for soft-assembly can be improved further. Specifically, while combining changes in dimensionality with changes in fractal scaling is novel, and both reflect necessary conditions of soft-assembly, future work can explore additional markers. For example, we would also expect changes in lacunarity (Plotnick et al., 1996), which differentiates the spatial patterns of fractals, and further, that different models of change should characterize the observed temporal patterns the system exhibits (e.g., oscillations). Future research can also build upon the present results by examining a larger variety of tasks and connecting the behaviors with their associated brain states. This may include investigating familiarity effects more exhaustively, as well as by developing more temporally precise measures. Finally, whereas soft-assembly logic dictates dimensional compression and a more ordered system following the transition to a task state, much more work can be done to characterize how factors such as cognitive load, task complexity, context, and individual differences moderate task-related change in measures.

## Conclusion

In short, our results suggest that dimensionality estimates and fractal scaling exponents systematically change during the transition from the spontaneous non-task related (“resting”) EEG state to that of the task-related pre-stimulus period and the post-stimulus ERP. These findings satisfy some of the necessary and initial conditions for establishing that human brain activity exhibits soft-assembly. That is, the notion that neural networks can fluidly and flexibly coordinate to function effectively. We envision that the conceptual framework provided by soft assembly, and its associated analytic approaches, may help to augment present approaches to characterizing task-related behavioral and neurophysiological change. In turn, this knowledge can further our understanding of the ways in which the highly-dimensional human system is able to functionally and flexibly coordinate so many components to meet environmental demands.

## Author contributions

ME and TM designed the experiment, and collected and processed the data. TW and TM conducted the analyses. All authors conceived and wrote the manuscript and approved it for publication.

### Conflict of interest statement

The authors declare that the research was conducted in the absence of any commercial or financial relationships that could be construed as a potential conflict of interest.

## Footnotes

^1^We originally estimated the scaling exponents up to the maximum possible window size of 1,024. However, based on discussion with one of our reviewers, the max 256 (*N*/4) window size was deemed more suitable given the relatively short length of the time series (Peng et al., 1994, 1995a). The overall pattern of results was the same, with the exception that there was a significant familiarity effect that was relatively small and ceased to persist with the modified max window size. Therefore, we view the results in their present form as more conservative than the initial analyses.

^2^These *post-hoc* analyses were developed and included based on important issues raised in the review process.
